# Two complete mitochondrial genomes of *Papilio* butterflies obtained from historical specimens (Lepidoptera: Papilionidae)

**DOI:** 10.1080/23802359.2021.1909443

**Published:** 2021-04-05

**Authors:** Ming-Luen Jeng, Ming-Yu Chen, Li-Wei Wu

**Affiliations:** a Department of Biology, National Museum of Natural Science, Taichung, Taiwan, ROC; b Department of Life Science, Tunghai University, Taichung, Taiwan, ROC

**Keywords:** Meta-genomics, mitogenomic phylogeny, *Pterourus*, *Chilasa*, *Heraclides*

## Abstract

Museum specimens are collected for education, exhibition, and various multiple scientific purposes. However, millions of specimens remain in their collection boxes for years without being analyzed. Historical specimens have been known to contain low-quality DNA; hence, it is difficult to utilize their sequence information in phylogenetic studies. However, recent advances in high-throughput sequencing (HTS) make these collections amenable to phylogenomic studies. In this study, two historical specimens (*Papilio xuthus* Linnaeus, 1767, and *Papilio thoas* Linnaeus, 1771) were sampled and DNA extracted for HTS *via* the Miseq platform. Two complete mitogenomes were assembled, even though the DNA quality of those specimens was highly fragmented, below 250 bp in length. The 37 genes of 60 mitogenomes were aligned and used for inferring the phylogenetic relationships of Papilioninae. These two newly sequenced mitogenomes are correctly grouped in the genus *Papilio*, and this result indicates that historical specimens show great potential for phylogenetic studies with HTS technology.

Swallowtail butterflies (Lepidoptera: Papilionidae) are insects of interest, with large size, and diverse wing patterns that make them iconic for insect conservation in Taiwan. Papilionidae emerged at the end of Cretaceous (Allio et al. 2020), and currently three subfamilies containing seven tribes are categorized. The animal mitochondrial genome is around 16 Kb in length (Boore 1999). Interestingly, the mitogenomic phylogeny of Papilionidae (Condamine et al. 2018) presented similar phylogenetic relationships and dating schemes as the genomic phylogeny as a whole (Allio et al. 2020). Meanwhile, a similar pattern is also found in skipper butterflies (Li et al. 2019), indicating that mitogenomic sequences are excellent markers to reconstruct butterfly relationships.

Taiwan is known as the Butterfly Kingdom for its great butterfly diversity (about 400 species), and its flourishing butterfly industry between the 1950s–1970s. At the peak of this industry, a significant number of imported butterflies were shipped to Taiwan from all over the world to create specimens as well as illustration until CITES was activated in 1975 (Unno 1974; Severinghaus 1977; Marshall 1982; Hamano 1987; New 1987). After the decline of the industry in the 1980s, some traders donated their remaining materials to local museums. For example over 100,000 butterfly specimens were donated to the National Museum of Natural Science, Taiwan (NMNS) by the family of Mr. Sheng-Keng Wang in 2010.

The collection contains diverse butterflies from all over the world, including many *Papilio* and birdwing specimens. The possibility of cross examining the genetic variation and the other molecular features which are vital to research and conservation is compelling, so we set a goal to explore the possibility of using these specimens for molecular studies. Two species were selected for high-throughput sequencing (HTS). The first one, *Papilio thoas* (voucher code: 16LW03015), is the type species of the subgenus *Heraclides*, and no public mitogenome could be obtained from GenBank. The other is *Papilio xuthus* (voucher code: 16LW03016), which is a very common butterfly distributed throughout East Eurasia. Three complete or partial mitogenomes have been deposited in a public database (NC_029244, KU356933, and EF621724). These two specimens do not have collection data, but they are estimated to have been kept in Wang’s collection more than 50 years. Two legs of the specimens were used to extract the genomic DNA, following the molecular processes of previous work (Chen et al. 2018), but only 10 μL sterile water were added in order to ensure a high concentration. The DNA concentrations were measured using the Qubit dsDNA HS Assay kit (Thermo Fisher Scientific, Waltham, MA), and the extracts were 1.6 ng/μL (*P. thoas*) and 18.1 ng/μL (*P. xuthus*), respectively. All the genomic DNAs were used directly for HTS library construction using the NuGEN Ovation Ultralow Library System (NuGEN Technologies, San Carlos, CA) without any further DNA fragmentation *via* the Miseq platform. After removing low DNA quality regions (below Q20), 1,702,186 (*P. thoas*; average length, 219 bp) and 3,428,700 (*P. xuthus*; average length, 223 bp) reads were obtained. The Lepidopteran mitogenomic dataset (Chen et al. 2018) were used to filter out the mitochondrion-like sequences of these samples, using CLC Genomics Workbench version 9 (CLC bio, Aarhus, Denmark). Then the mapping reads were *do novo* assembled with 97% similarity using CLC Genomics Workbench and megahit (Li et al. 2015). The contigs of these two mitogenomes were checked, edited and combined using Sequencher version 4.10 (GeneCode, Boston, MA).

Gene regions and annotation were predicted using MITOS2 (Bernt et al. 2013), but the reference mitogenome, *Papilio maraho* (Wu et al. 2010), was used to confirm their gene regions. As a result, the two complete mitogenomes of *P. thoas* (MW548255; 15258 bps, average coverage: 116–594) and *P. xuthus* (MW548256; 15350 bps, average coverage: 1210–3959) were produced, containing 37 mitochondrial genes (13 protein-coding genes, 22 transfer *RNA*s, and two ribosomal *RNA*s) with standard gene order as with other butterflies.

Total of 60 Papilionidae butterflies were used to infer phylogenetic relationships (Figure 1). The 37-gene dataset is 15,725 bp in length, and the prior partitions were set to each gene. The maximum likelihood (ML) tree was reconstructed using IQ-TREE (Nguyen et al. 2015), and the nodal supports were evaluated by 1000 replicates of bootstrapping. Overall, the phylogenetic positions of this Papilioninae phylogeny are in concordance with the topology of a previous effort (Condamine et al. 2018). These two species are both grouped in the tribe Papilionini. *Papilio thoas* presents sister lineage to other *Papilio* butterflies (Allio et al. 2021), whereas four *P. xuthus* are grouped together, sister to *P. machaon*. Our results demonstrated that with the HTS technology, extracting DNA data from historical specimens warrants more investigation and further studies of mitogenomic sequences in Wang’s collection are recommended.

**Figure 1.**
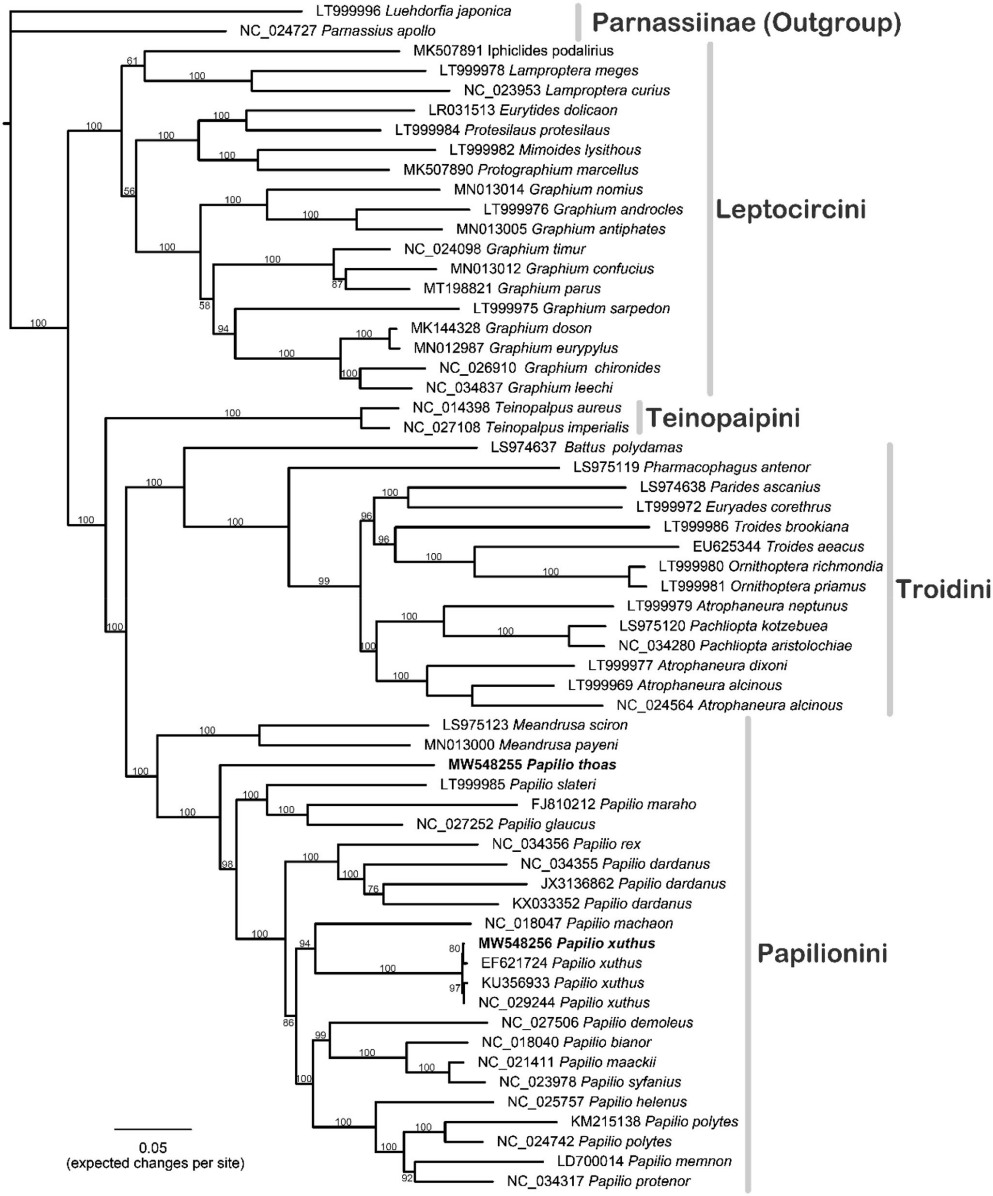
The ML phylogeny of the Papilioninae based on 60 mitogenomes using IQ-tree. The Parnassiinae butterflies (*Luehdorfia japonica* and *Parnassius apollo*) were set as outgroups, and the total aligned length (37 genes) is 15,725 bp.

## Data Availability

The data that support the findings of this study are openly available in National Center for Biotechnology Information (NCBI) at https://www.ncbi.nlm.nih.gov/nucleotide/, reference numbers MW548255 and MW548256.
